# Deep Immunophenotyping of Circulating T and B Cells in Relapsing Adult-Onset Still’s Disease

**DOI:** 10.3390/cimb46020075

**Published:** 2024-02-01

**Authors:** Valentina Myachikova, Igor Kudryavtsev, Artem Rubinstein, Arthur Aquino, Dmitry Isakov, Alexey Golovkin, Alexey Maslyanskiy

**Affiliations:** 1 Rheumatology and Immunopathology Research Laboratory, Federal State Budgetary Institution “Almazov National Medical Research Centre” of the Ministry of Health of the Russian Federation, 197341 St. Petersburg, Russia; myachikova_vyu@almazovcentre.ru (V.M.);; 2 Autoimmune and Autoinflammatory Diseases Research Laboratory, Federal State Budgetary Institution “Almazov National Medical Research Centre” of the Ministry of Health of the Russian Federation, 197341 St. Petersburg, Russia; 3 Laboratory of Cellular Immunology, Institute of Experimental Medicine, 197376 St. Petersburg, Russia; 4 Department of Immunology, First St. Petersburg State Medical University, 197022 St. Petersburg, Russia; 5 Scientific, Clinical and Educational Centre of Gastroenterology and Hepatology, Saint Petersburg State University, 199034 St. Petersburg, Russia

**Keywords:** adult-onset Still’s disease, AOSD, autoinflammation, ‘IL-1 driven’ disease, Th cells, CD8+ T cells, B cells, flow cytometry

## Abstract

Adult-onset Still’s disease (AOSD) is a complex systemic inflammatory disorder, categorized as an ‘IL-1 driven’ inflammasomapathy. Despite this, the interaction between T and B cells remains poorly understood. We conducted a study, enrolling 7 patients with relapsing AOSD and 15 healthy control subjects, utilizing deep flow cytometry analysis to examine peripheral blood T- and B-cell subsets. T-cell and B-cell subsets were significantly altered in patients with AOSD. Within CD4+ T cells, Th2 cells were decreased. Additionally, Th17 cell and follicular Th cell subsets were altered within CD45RA–CD62L+ and CD45RA–CD62L– Th cells in patients with AOSD compared to healthy controls. We identified changes in CD8+ T cell maturation and ‘polarization’ in AOSD patients, with an elevated presence of the TEMRA CD8+ T cell subset. Furthermore, the percentage of Tc1 cells was decreased, while the frequency of CCR6–CXCR3– Tc2 cells was elevated. Finally, we determined that the frequency of CD5+CD27– B cells was dramatically decreased in patients with AOSD compared to healthy controls. Further investigations on a large group of patients with AOSD are required to evaluate these adaptive immunity cells in the disease pathogenesis.

## 1. Introduction

Still’s disease, initially described by Sir George Frederic Still in children [1], was renamed in 2001 as systemic juvenile idiopathic arthritis (sJIA) for standardized terminology. Eric Bywaters identified it in adults 75 years after the first description [2], labeling it adult-onset Still’s disease (AOSD). Until 2014, AOSD and sJIA were regarded as distinct nosologies, but this perception changed following gene expression analysis in 2014 [3]. This rare disorder has an annual incidence of 0.16 cases per 100,000 persons, marked by sterile inflammation, prolonged fever, musculoskeletal involvement, skin manifestations, serous membrane involvement, other variable symptoms, and prominent neutrophil-driven leukocytosis [4].

The systemic polycyclic course of AOSD is recognized for its distinct phases of relapse and remission. During a relapse, patients experience severe symptoms, marked by abnormal laboratory test results, showcasing a diverse array of pro-inflammatory cytokines and acute-phase reactants, particularly elevated levels of ferritin. Elevated ferritin concentrations independently predict the emergence of secondary macrophage-activating syndrome (MAS) or secondary hemophagocytic lymphohistiocytosis (HLH) [5]. Conversely, in remission, symptoms abate, and laboratory indicators revert to normal levels [6].

The etiology remains unknown, and the pathogenesis, considered a possible mixed (autoinflammatory > autoimmune) systemic inflammatory disease, involves the dysregulation of both the innate and adaptive immune systems [7]. On the one hand, gathered data suggest dysregulation within the innate immune system, reflecting the autoinflammatory nature of the disease, characterized by the activation of interleukin-1 beta (IL-1β) and interferon-gamma (IFN-γ) signaling pathways [8].

Studies have shown increased nucleotide-binding oligomerization domain (NOD)-and leucine-rich repeat (LRR)-containing receptor pyrin domain-containing-3 (NLRP3) expression in peripheral blood mononuclear cells (PBMCs) from AOSD patients that positively correlates with disease activity [9]. On the other hand, a number of patients exhibit a genetic association with major histocompatibility complex (MHC) class II alleles [7], highlighting the potential influence of adaptive immunity (indicating an autoimmune origin of the disorder). This observation may elucidate why not all patients respond to therapy with IL-1 and IL-6 blockers, as well as the transition from a relapsing to a chronic course.

Limited studies explore adaptive immunity’s role in Still’s disease pathogenesis. Prolonged proinflammatory cytokine synthesis, in contrast to monogenic periodic fevers, may significantly impact not only on visceral organs but also secondary lymphoid tissues where T- and B-lymphocyte differentiation underlying acquired immunity reactions occurs. Specifically, IL-1β and IL-18 contribute to Th0 polarization toward Th1 and Th17 cell fates in lymphoid tissue [10]. IL-1β increases the number of IL-17 and IFN-γ-producing cells, granzyme B-expressing cells, IFN-γ-producing cytolytic CD8+ T cells [11], and, in conjunction with IL-6 and IL-23, it promotes Th17-skewed CD4+ T-cell differentiation [12].

This study aimed to analyze the peripheral blood immune cell profile by using flow cytometry to gain deeper insights into immune system functioning during relapsing AOSD.

## 2. Materials and Methods

### 2.1. Patient Characteristics

From April to October 2022, we enrolled 7 patients with a relapse of AOSD in this study. All patients met the diagnostic criteria for AOSD, as proposed by Yamaguchi et al. [13]. Disease activity was assessed using the modified Pouchot’s score (by Rau M.) [14]. We documented the clinical data during the relapse (not all symptoms the patients had previously) (Table 1) and collected whole blood samples from all participants after an overnight fast.

Six patients experienced a severe exacerbation of the disease, while one (Patient 7, Table 1) had a moderate relapse with inadequate pathogenic treatment (4 patients were on a low dose of NSAIDs, and 3 patients were on a low dose of prednisolone (with or without methotrexate), as indicated in Table 1). Additionally, the concentration of C-reactive protein was elevated in all patients. Six patients had a polycyclic course of the disease with a prolonged medical history, whereas one patient had a monocyclic course. All patient characteristics are presented in Table 1. Control group consisted of 15 age-matched apparently healthy controls (HCs) (5 men and 10 women) in this study. This study was conducted in accordance with the Declaration of Helsinki, and approved by the local Ethics Committee of the Almazov National Medical Research Center (protocol code 28, 12 February 2018). All participants provided written informed consent.

### 2.2. Sample Collection

Peripheral blood was collected into vacuum K3-EDTA-containing test tubes and was then processed to analyze less than 6 h after collection. The frequencies of the main circulating T- and B-cell subsets were analyzed using flow cytometry. CytoFlex S Flow Cytometer (Beckman Coulter, Indianapolis, IN, USA) was used for immunophenotyping.

### 2.3. CD3+ T-Cell Phenotyping via Flow Cytometry

T-cell surface markers in peripheral blood samples were detected by flow cytometric analysis. For CD4+ T-cell subsets, peripheral blood samples (200 μL) were incubated with anti-human CD45RA-FITC, CD62L-PE, CXCR5-PerCP/Cy5.5, CCR6-PE/Cy7, CXCR3-APC, CD3-APC/Cy7, CD4-PacBlue, and CCR4-BV510. The detailed characteristics of antibodies are given in Supplementary Table S1. Gaiting strategy was described previously in detail [14]. For CD8+ T-cell subsets, CD4-PacBlue was replaced with CD8-PacBlue. The detailed characteristics of antibodies are given in Supplementary Table S2. All antibodies were manufactured by BioLegend, Inc. (San Diego, CA, USA) and were used according to the manufacturer’s recommendations. Gaiting strategies were described previously in detail [15] and are shown in Supplementary Figures S1 and S2.

Blood samples were incubated with antibodies for 15 min in the dark at room temperature. For red blood, cells were lysed for 15 min in the dark with 2 mL of the VersaLyse Lysing Solution (Beckman Coulter, Inc., Indianapolis, IN, USA) supplied with 50 μL of the IOTest 3 Fixative Solution (Beckman Coulter, Inc., Indianapolis, IN, USA). Next, all samples were washed for 7 min at 330 g with 4 mL of a sterile phosphate-buffered saline (PBS) supplied with 2% of heat-inactivated fetal bovine serum (Sigma-Aldrich, St. Louis, MO, USA). Finally, leukocytes were resuspended in 0.5 mL PBS containing a 2% neutral-buffered formalin solution (Sigma-Aldrich, St. Louis, MO, USA) and analyzed via flow cytometry. At least 40,000 CD4+ T cells or 20,000 CD8+ T cells were analyzed in each sample.

### 2.4. CD19+ B-Cell Phenotyping via Flow Cytometry

B-cell subset frequencies and phenotypes in the peripheral blood samples from patients with AOSD and healthy controls were analyzed through flow cytometry. In brief, 100 μL of whole peripheral blood was stained with the following cocktail of mouse anti-human antibodies: IgD-Alexa Fluor 488, CD38-PE, CD27-PE/Cy7, CD24-APC, CD19-APC/Cy7, CD5-PacBlue, and CD45-KrO. The detailed characteristics of antibodies are given in Supplementary Table S3. Red blood cell lysing procedure was followed by cell washing, as described above. At least 5000 CD19+ B cells were analyzed for each sample. The gating strategy was described previously [16] and is shown in Supplementary Figure S3.

### 2.5. Statistical Analysis

The flow cytometry data analysis was performed using Kaluza software v2.1 (Beckman Coulter, Indianapolis, IN, USA). Statistical calculations were performed using GraphPad Prism 8 (GraphPad software Inc., San Diego, CA, USA) and Statistica 7.0 (StatSoft, Tulsa, OK, USA) software. Normality was checked using Pearson’s chi-squared test. All data are presented as a percentage of cells in the main T- or B-cell subsets. Quantitative data are expressed as median and interquartile range: Me (25; 75) in all tables and figures. Nonparametric Mann–Whitney U-test was used to compare different groups. Significance was set at *p* < 0.05.

## 3. Results

### 3.1. Alterations in Main Circulating Lymphocyte Subsets in Patients with AOSD

We performed flow cytometry to determine the percentages of CD3+, CD19+, CD8+, and CD4+ T cells in the blood samples of 7 patients with AOSD and 15 patients from the control group. We noticed that patients with AOSD had increased frequencies of T cells (81.08% (76.96; 87.78) vs. 72.53% (68.57; 73.88) with *p* = 0.007), while CD19+ B cells were decreased (5.32% (4.06; 9.97) vs. 12.54% (8.16; 16.13) with *p* = 0.018, respectively). Furthermore, in peripheral blood samples from the AOSD group, the levels of CD8+ T cells were elevated if compared to healthy controls (30.77% (26.44; 39.60) vs. 23.42% (19.55; 28.52), *p* = 0.010), but these two groups had similar percentages of CD4+ T cells (47.09% (30.05; 57.17) vs. 47.04% (40.65; 47.94), respectively, with *p* = 0.805) and similar CD4/CD8 ratio (1.53 (0.87; 2.17) vs. 1.77 (1.52; 2.44), respectively, with *p* = 0.192).

### 3.2. Alterations in Th Cell Maturation and ‘Polarization’ in Patients with AOSD

**Figure 1:**
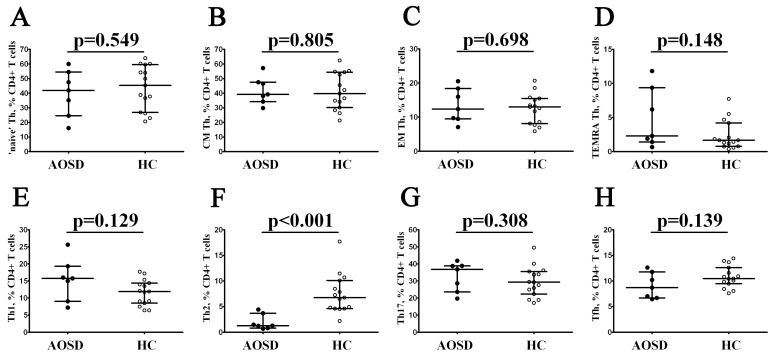
Alterations in Th cell maturation and ‘polarization’ in patients with AOSD. Scatter plots (**A**–**D**) show the frequencies of ‘naïve’, central memory (CM), effector memory (EM), and terminally differentiated CD45RA-positive effector memory (TEMRA) CD4+ T cells within total CD4+ T cells, respectively. Scatter plots (**E**–**H**) show Th1, Th2, Th17, and follicular Th frequencies within total CD4+ T cells, respectively. Black circles—patients with AOSD (AOSD, n = 7); white circles—healthy control (HC, n = 15). Each dot on scatter plots represents individual subjects, and horizontal bars show the group medians and quartile ranges (Med (Q25; Q75)). The *p*-value was determined by the Mann–Whitney U-test.

Primarily, we identified main maturation Th subsets classified by CD45RA and CD62L co-expression (Figure 1A–D), and no significant differences were found in the ‘naïve’ (CD45RA+CD62L+), central memory (CD45RA–CD62L+), effector memory (CD45RA–CD62L–), or TEMRA (CD45RA+CD62L–) Th cell frequencies between patients with the AOSD and control groups. Next, to assess relevant circulating ‘polarized’ CD4+ T cell subsets, we studied the cell surface co-expression of CXCR5, CXCR3, CCR4, and CCR6 chemokine receptors on peripheral blood Th cells, as described previously [14]. Th1 cells were identified as CXCR5–CXCR3+CCR4–CCR6–, Th2 cells had CXCR5–CXCR3–CCR4+CCR6– phenotype, Th17 cells were CXCR5–CCR6+, and follicular Th cells expressed CXCR5 on their cell membrane. We noticed (Figure 1E–H) that patients with AOSD had dramatically decreased numbers of CXCR5–CXCR3–CCR4+CCR6– Th2 cells within the total CD4+ T-cell subset if compared to the healthy control group (1.29% (0.81; 3.70) vs. 6.77% (4.62; 10.11) with *p* < 0.001).

**Figure 2:**
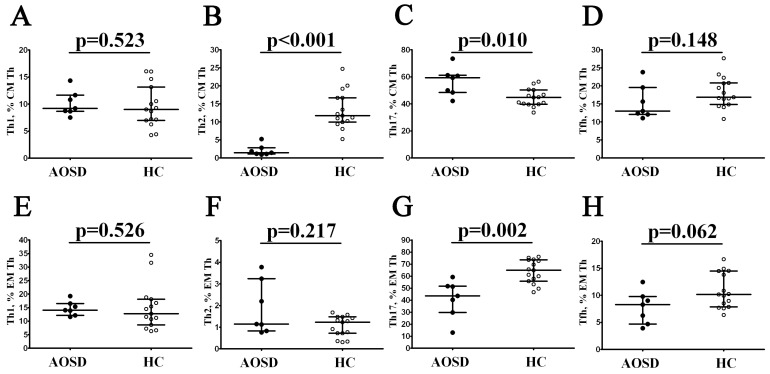
Alterations in relative numbers of Th2 and Th17 cell subsets in patients with AOSD. Scatter plots (**A**–**H**) show the frequencies of Th1, Th2, Th17, and follicular Th within total CM and EM CD4+ T cells, respectively. Black circles denote patients with AOSD (AOSD, n = 7); white circles—healthy control (HC, n = 15). Each dot on scatter plots represents individual subjects, and horizontal bars show the group medians and quartile ranges (Med (Q25; Q75)). The *p*-value was determined by the Mann–Whitney U-test.

We next assessed the level of ‘polarized’ Th cell subsets within central memory cells that patrol secondary lymphoid organs, and effector memory cells, able to migrate to the peripheral inflamed tissue to augment the immune responses. As shown in Figure 2, within central memory Th cells, patients with AOSD had increased frequencies of Th17 cells, while Th2 cells were decreased when compared to the HC group (59.41% (48.46; 61.26) vs. 44.88% (39.78; 50.32) with *p* = 0.001 and 1.48% (1.11% (2.86) vs. 11.76% (9.95; 16.69) with *p* < 0.001, respectively). Oppositely, Th17 levels in patients with AOSD were decreased within effector memory Th cells when compared to healthy controls (43.58% (29.82; 51.62) vs. 64.99% (55.69; 73.49), *p* = 0.002). We also indicated that these two groups had similar percentages of Th1 and follicular Th cells within CM and EM CD4+ T-cell subsets.

Next, because patients with AOSD had an altered distribution of peripheral blood CM and EM Th17 cells, we investigated changes in distinct Th17 subsets with different patterns on CXCR3 and CCR4 expression. Currently, there are four major Th17 cell subsets, including CCR4+CXCR3– ‘classical’ Th17 cells, CCR4+CXCR3+ double-positive (DP) Th17 cells, CCR4–CXCR3+ ‘non-classical’ or Th17.1 cells, and CCR4–CXCR3– double-negative (DN) Th17 [17,18]. We noticed that patients with AOSD showed dramatically elevated levels of DN Th17 cells within CM CCR6+ Th cells but decreased frequencies of ‘classical’ and DP Th17 cells if compared to the healthy control group (Table 2). Next, we found increased levels of DN Th17 and ‘classical’ Th17 cells within CCR6+ EM CD4+ T cells, while the numbers of non-classical Th17.1 cells were decreased if compared to HC.

Finally, we focused on circulating CXCR5-expressing follicular Th cell subsets and identified four maim Tfh cell subsets, including CXCR3+CCR6– Tfh1, double-negative CXCR3–CCR6–Tfh2, CXCR3–CCR6+ Tfh17, and double-positive CXCR3+CCR6+ DP Tfh cells, based on CXCR3 and CCR6 chemokine receptor co-expression [19]. First, we noticed a decreased frequency of central memory Tfh17 cells in patients with AOSD (24.63% (21.75; 27.81) vs. 33.64% (27.26; 37.29) with *p* = 0.008, Table 3). Next, within CD45RA–CD62L– effector memory Tfh cells that were able to leave circulating blood, to migrate to the sites of inflammation and to take part in ectopic lymphoid structure formation [20], we found decreased levels of Tfh2 cells (6.94 (1.57; 11.70) vs. 14.23 (9.89; 18.58) with *p* = 0.010) and increased levels of DP Tfh cells (31.53 (22.40; 37.80) vs. 18.86 (17.00; 23.48) with *p* = 0.007) in patients with AOSD vs. healthy controls.

### 3.3. Alterations in CD8+ T-Cell Maturation and ‘Polarization’ in Patients with AOSD

**Figure 3:**
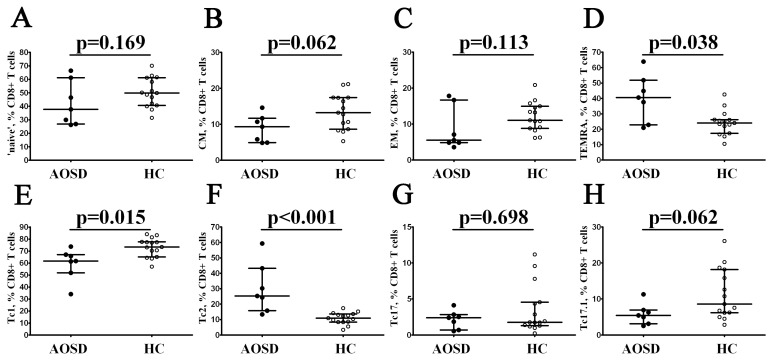
Alterations in CD8+ T-cell maturation and ‘polarization’ in patients with AOSD. Scatter plots (**A**–**D**) show the percentages of ‘naïve’, central memory (CM), effector memory (EM), and TEMRA CD8+ T cells within total CD8+ T cells, respectively. Scatter plots (**E**–**H**) show Tc1, Tc2, Tc17, and Tc17.1 cells, respectively. Black circles denote patients with AOSD (AOSD, n = 7); white circles—healthy control (HC, n = 15). Each dot represents individual subjects, and horizontal bars depict the group medians and quartile ranges (Med (Q25; Q75)). The *p*-value was determined by the Mann–Whitney U-test.

As shown previously, we found increased numbers of circulating CD8+ T cells in patients with AOSD. Primarily, using multicolor flow cytometry, we assessed the frequencies of peripheral blood CD8+ T-cell subsets with different CD45RA and CD62L co-expression. Similar to CD4+ T cells, we were able to subdivide CD8+ T cells into naïve, central memory, effector memory, and TEMRA cells (Figure 3). We found that the relative number of the most mature CD8+ T-cell subset—TEMRA—was elevated in patients with AOSD if compared to healthy controls (40.51% (22.85; 51.81) vs. 24.06% (17.33; 26.26), *p* = 0.038). Next, we analyzed CXCR3 and CCR4 co-expression on the cell surface of CD8+ T cells. Within total CD8+ T cells, we identified CCR6–CXCR3+ Tc1 cells, CCR6–CXCR3– Tc2 cells, CCR6+CXCR3– Tc17, and double-positive CCR6+CXCR3+ Tc17.1, as proposed earlier [21,22]. While examining ‘polarized’ CD8+ T-cell counts, it was found that in patients with AOSD, the percentage of Tc1 cells was decreased (61.72% (51.88; 66.90) vs. 73.31% (65.09; 77.59) with *p* = 0.015). Moreover, the frequency of CCR6–CXCR3– Tc2 cells was elevated (25.24% (15.73; 43.32) in the AOSD group vs. 10.92% (8.34; 13.60) in the control group with *p* < 0.001).

**Figure 4:**
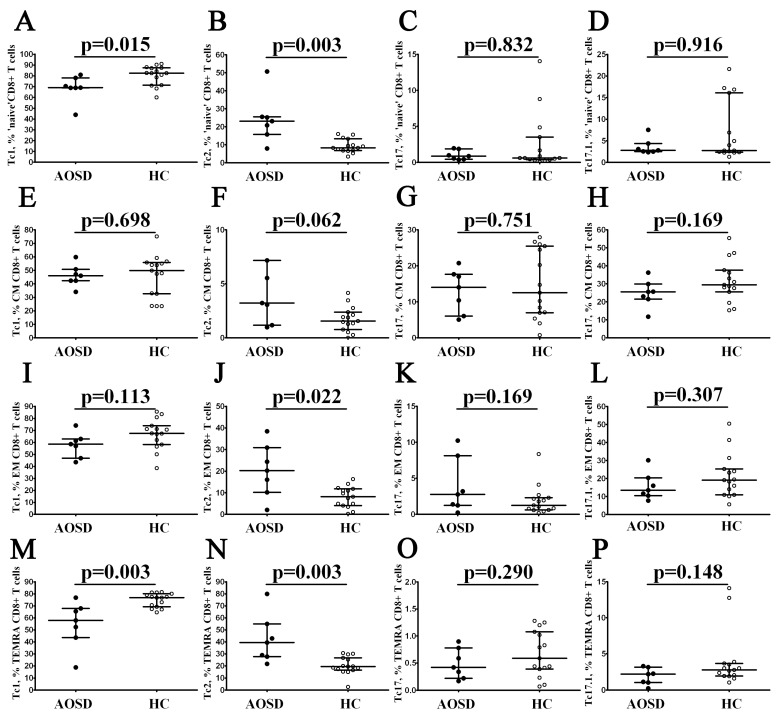
Imbalance in peripheral blood Tc1, Tc2, Tc17, and Tc17.1 cells in major CD8+ T-cell subsets with varying patterns of CD45RA and CD62L expression in patients with AOSD. Scatter plots (**A**–**P**) show the relative numbers of Tc1, Tc2, Tc17 andTc17.1 cells within ‘naïve’, central memory, effector memory, and TEMRA CD8+ T cells, respectively. Black circles denote patients with AOSD (AOSD, n = 7); white circles—healthy control (HC, n = 15). Each dot represents individual subjects, and horizontal bars depict the group medians and quartile ranges (Med (Q25; Q75)). The *p*-value was determined by the Mann–Whitney U-test.

Next, we analyzed the frequency of CCR6–CXCR3+ Tc1 within ‘naïve’, CM, EM, and TEMRA CD8+ T cells in patients with AOSD (Figure 4) and found that the level of Tc1 cells in ‘naïve’ and TEMRA CD8+ T cells was decreased if compared to healthy controls (69.01% (68.79; 78.10) vs. 82.54% (71.41; 87.36) with *p* = 0.015 for ‘naïve’ CD8+ T cells and 57.87% (43.47; 67.76) vs. 76.89% (69.22; 80.09) with *p* = 0.003 fro TEMRA CD8+ T cells, respectively). Next, compared with the healthy control, the percentages of CCR6–CXCR3– Tc2 cells in patients with AOSD were increased in ‘naïve’ EM and TEMRA CD8+ T cells (23.18% (15.82; 25.62) vs. 8.37% (6.82; 13.44) with *p* = 0.003, 20.25% (10.23; 30.89) vs. 8.18% (4.08; 11.82) with *p* = 0.022, and 39.43% (27.73; 54.96) vs. 19.32% (16.45; 26.65) with *p* = 0.003, respectively). Nevertheless, we found no significant differences in frequencies of CCR6-expressing CD8+ T-cell subsets between patients with AOSD and healthy controls.

### 3.4. Alterations in CD19+ B-Cell Maturation in Patients with AOSD

Finally, considering the potential role of specific humoral immunity alterations during adult-onset Still’s disease, we focused on circulating B cells and their main subsets. Primarily, for B-cell subset immunophenotyping, we used the so-called “Bm1-Bm5” classification, which is based on the relative expression of IgD and CD38 [23]. We revealed six main B-cell maturation stages, including “naïve” IgD+CD38– or Bm1 cells, “activated naïve” IgD+CD38+ or Bm2 cells, ‘pre-germinal-center’ IgD+CD38++ or Bm2 cells, so-called IgD-CD38++ “Bm3 + Bm4” cell subset, that contained containing centroblasts and centrocytes, and peripheral blood circulating memory subsets, early memory IgD–CD38+ or eBm5 cells, and resting memory IgD–CD38– cells or Bm5 cells. As shown in Table 4, we found that patients with AOSD had decreased levels of Bm2 cells and increased frequencies of resting memory Bm5 cells.

**Figure 5:**
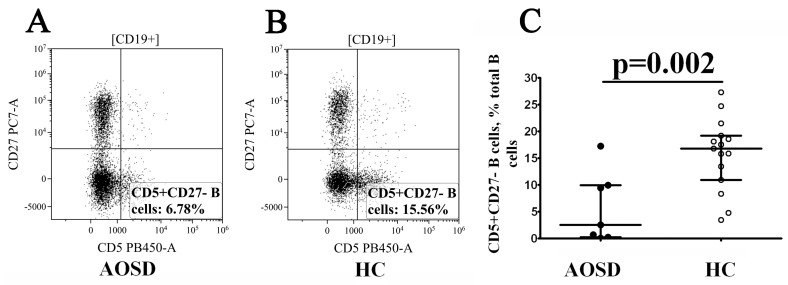
Decreased relative number of CD5+CD27– B cells in patients with AOSD. Representative dot plots showing expression of CD5 vs. CD27 in a patient with AOSD (**A**) and healthy control subject (**B**). Scatter plots (**C**) show the relative number of CD5+CD27– cells among total B-cell subset, respectively, in the peripheral blood samples from patients with AOSD (n = 7, black circles, AOSD) and healthy controls (n = 15, white circles, HC). Each dot represents individual subjects, and horizontal bars represent the group medians and quartile ranges (Med (Q25; Q75). The *p*-value was determined by the Mann–Whitney U-test.

Finally, we determined CD5 vs. CD27 co-expression and distinguished CD5+CD27– cells with regulatory properties within the total B-cell population. We found that the frequency of CD5+CD27– B cells was dramatically decreased in patients with AOSD vs. healthy controls (2.53% (0.24; 9.98) vs. 16.80% (10.92; 19.21) with *p* = 0.002, Figure 5).

## 4. Discussion

Here, we present the study data, attempting to analyze the circulating adaptive immune lymphocyte profile in AOSD patients by assessing the phenotypic characteristics of CD4+ and CD8+ T cells as well as CD19+ B cells, by focusing on the relevant maturation and ‘polarization’ status.

The data obtained suggest that the Th cell ratio underwent profound alterations, both within central memory cells, mirroring events occurring in lymphoid tissues, and in effector memory Th cells, regulating the functional activity of effector cells regarding the inflammatory focus via produced effector cytokines. For instance, a higher percentage of CM Th17 cells indicates that their elevated generation in lymphoid tissues may be related to the high level of “polarizing” Th17 cytokines in patients with this pathology.

In this regard, it was previously shown that an upregulated level of NLRP3 inflammasome and its correlation with disease activity evidence that activated NLRP3 is involved in AOSD pathogenesis [4]. The hyperactivation of the NLRP3 inflammasome subunit may result in the hyperproduction of active proinflammatory IL-1 family cytokines, primarily IL-1β and IL-18 [6,24]. In turn, these cytokines can elicit the activation of other downstream proinflammatory cytokines, such as IL-6, IL-8, IL-17, and tumor necrosis factor alpha (TNF-α), involved in AOSD pathogenesis and triggering events related to induced inflammation and T cell “polarization” [25,26]. Moreover, the relative abundance of IL-17-secreting (Th17) cells in patients with active AOSD vs. control subjects was increased [27]. For instance, the peripheral blood Th17 cell level correlated with disease activity, ferritin levels as well as concentrations of serum cytokines, such as IL-1β, IL-6, IL-17, IL-18, IL-21, and IL-23. On the other hand, upon reaching the clinical remission phase, the peripheral blood level of Th17 cells and IL-17 as well as ferritin decreased compared with active AOSD. Our personal data, showing a decline in the CM Th17 cell level, indirectly corroborate that Th17 cells are specifically recruited to peripheral inflammatory foci.

It should also be noted that we revealed prominent alterations in Th17 cell subset composition, associated primarily with a lower percentage of CXCR3-expressing Th17 cells putatively due to their targeted recruitment of a gradient of CXCR3 ligands—CXCL9, CXCL10, and CXCL11—to the site of inflammation. It should be mentioned that the serum level of pro-inflammatory chemokine CXCL10 in AOSD was markedly higher than in patients with RA and control subjects [28]. In addition, a strong relation between the CXCL10 level and validated markers of disease activity, including ferritin and AST as well as systemic assessment scores, was observed. Most patients with AOSD later experienced profoundly decreased CXCL10 levels after effective therapy, although a change in CXCL10 levels correlated solely with changed systemic parameters.

Moreover, CXCL10 and its cognate receptor CXCR3 were highly expressed in skin lesions, whereas increased CXCL10 accumulation was observed in inflammatory cells along with mucin deposits. Moreover, another study showed that apart from IFN-γ, the level of all three CXCR3 ligands—CXCL9, CXCL10, and CXCL11—was increased in the peripheral blood of AOSD patients [29]. An increase in the level of such chemokines was closely related to the elevated amount of key peripheral blood inflammatory factors—C-reactive protein and ferritin. Moreover, the accumulation of CD4+ T cells at inflammatory sites was tightly linked to CXCL11 production.

Moreover, we found a prominent decline in the CM Th2 cell level in AOSD patients vs. control subjects, which also affected the Th1/Th2 cell ratio, which was skewed to a higher percentage of Th1 lymphocytes. It should be noted that in the active phase, IL-4 and IL-13, but not IFN-γ, IL-12, or IL-20 levels, were increased in patients with AOSD [30]. Moreover, serum IL-18 levels showed a good correlation with IL-4 levels, suggesting that AOSD is characterized by a Th2 rather than a Th1 cell cytokine profile. Our personal data also indicate that Th2 cells are actively involved in the pathogenesis of AOSD. On the other hand, new-onset AOSD is coupled to a higher level of circulating IFN-γ-producing Th1 cells paralleled with an increased Th1/Th2 cell ratio, verified by assessing the production of IFN-γ and IL-2, respectively [31]. Similar results were obtained while analyzing the gene expression levels of relevant cytokines in skin biopsy samples. Apart from this, the level of peripheral blood IFN-γ-producing Th1 cells was closely associated with a higher serum IL-18 level and AOSD clinical manifestations [31]. Three-month-long therapy with glucocorticoids and methotrexate revealed that patients with AOSD had decreased circulating IL-18 as well as peripheral blood IFN-γ+ Th1 cell levels.

We also observed a significantly altered profile in circulating CD8+ T cells, primarily manifested by their increased percentage in peripheral blood during AOSD, which is in line with the data obtained by Guo et al. [32]. Moreover, using cluster analysis, Guo et al. showed that patients with peak CD8+ T cell counts had maximum systemic scores at diagnosis and were more likely to have pulmonary infiltrates, pericarditis, splenomegaly, and poorer response to treatment as well as the highest total number of disease outbreaks. However, Jung et al. noted a significantly decreased CD8+ T cell level in AOSD compared to the control group [33]. It should also be noted that histological data in AOSD revealed an increased CD8+ T-cell count in the skin, liver, and bone marrow [34].

It is worth noting that we detected no changes in the CD8+ T-cell distribution across maturation stages. At the same time, some studies noted a higher percentage of peripheral blood “naïve” CD8+ T cells in AOSD [33]. On the other hand, a recent study by Chi et al. showed that during active AOSD, T cells are characterized by decreased levels of “naïve” cells and central memory cells, whereas the percentage of effector cells and effector memory cells increased [35]. Apparently, alterations in both CD4+ T cells and CD8+ T cell maturation are closely related to disease activity, which may underlie heterogeneity in the obtained data.

Moreover, while analyzing CD8+ T cells, we found a rise in the percentage of peripheral blood cytokine-producing Tc2 CD8+ T cells along with a decreased level of Tc1 cytolytic cells in patients with AOSD. It is also interesting to note that an inflamed lymph node biopsy from an AOSD patient revealed the dominance of CD8+ T cells, but only a small number of them expressed the maturity marker granzyme B [36] along with increased levels of Th2 cytokines, including IL-4 and IL-13 [30], which may also be related to an increased percentage of Tc2 cells at the systemic level. As noted above, patients with AOSD are characterized by high levels of CXCR3 ligands—CXCL9, CXCL10, and CXCL11—powerfully recruiting CXCR3-expressing cells (including Tc1 with CXCR3+CCR6– phenotype) into inflamed tissue [29]. Moreover, the synovial membrane level of TNF-α mRNA expression, one of the key effector cytokines in Tc1 cells, in AOSD was significantly increased compared with osteoarthritis but remained significantly lower than that observed in rheumatoid arthritis [24], allowing us to assume that some relevant cells may produce this cytokine in developing arthritis during AOSD.

Interestingly, an inflamed lymph node biopsy from an AOSD patient uncovered that CD8+ T cells prevailed, among which only a few expressed the T-cell maturity marker granzyme B [36]. At the same time, the accumulation of granzyme B is more typical for mature Tc1 CD8+ T cells able to migrate to the site of inflammation, where it may participate in IL-18 proform cleavage into the active pro-inflammatory counterpart by analogy with caspase-1 and mast cell chymase [37,38]. Therefore, our data as well as sparse publications suggest that CD8+ T cells might be involved in the pathogenesis of AOSD, which requires further research.

We also observed an altered CD19+ B-cell profile in AOSD, which is in line with previously published data [34,38]. We found that such alterations were associated with a higher percentage of resting memory IgD–CD38– B cells and decreased ‘pre-germinal-center’ Bm2 cell levels. Chi et al. observed, in active AOSD, a decline in the percentage of naïve and memory B cells, whereas the level of circulating plasma cell precursors was increased [35]. It should be noted that IL-1β promotes B-cell activation, proliferation as well as differentiation into plasma cells [39], which may be closely related to high levels of this cytokine in AOSD during increased disease activity. In contrast, Fang et al. showed that active AOSD is paralleled with an increased percentage of “naïve” IgD+CD27– B cells, double-negative IgD–CD27– B cells as well as CD20–CD27hi plasmablasts, whereas the “regulatory” B-cell subsets—IgD+CD27+ and CD24hiCD27+—significantly decreased [40].

Regarding B-cell subsets with regulatory properties, we noted a decline in the level of peripheral blood CD5-expressing B cells in Still’s disease. Hallmark studies point to the fact that CD5+ B cells can be found in diverse human tissues being able to produce autoantibodies (including rheumatoid factor and anti-ssDNA antibodies), as well as higher counts of CD5+ B cells in autoimmune diseases, such as rheumatoid arthritis and Sjögren’s syndrome [41,42]. Unfortunately, little is currently known about the functional characteristics of such cells and their role in the pathogenesis of human autoimmune diseases. Laboratory animal models uncovered that CD5-expressing B cells belong to the B1a population typically localized in the peritoneal cavity and produce low-affinity IgM antibodies with autoreactive specificity [43]. Moreover, IL-10 synthesized by CD5+ B cells is involved in controlling autoimmune reactions in murine experimental encephalomyelitis [44]. CD5 in humans is found on the cell membrane of transitional CD24+++CD38++ T1 B cells [45], but based on recent studies, such cells compared to other transitional B-cell subsets produce IL-10 at a relatively low level [46]. Interestingly, patients suffering from NLRP1-associated autoinflammatory syndrome (also characterized by high IL-18 and IL-1β levels) had higher peripheral blood transitional B cells, bearing the regulatory phenotype CD24highCD38highCD27low/neg [47]. Hence, various populations of human “regulatory” B cells remain poorly characterized; however, in the case of some autoinflammatory and autoimmune disorders, they can be used as diagnostic markers and potential therapeutic targets.

Finally, our findings provide new insights into the development of targeted therapies for patients with AOSD. Currently, the management of AOSD is predominantly based on anti-inflammatory therapy, rather than disease–modifying anti–rheumatic drugs (DMARDs). The concept of the “window of opportunity” for anti-cytokine therapy is extensively discussed, with a focus on IL-1 and IL-6 inhibitors, primarily as first-line agents. It has been observed that initiating this class of drugs early is more effective and leads to a more enduring response. In refractory cases and in the development of HLH, Janus kinase inhibitors as well as interferon-gamma inhibitors are also considered [7]. Furthermore, the efficacy of canakinumab therapy on patients with sJIA, both clinically and in the laboratory, was demonstrated in clinical trials, where gene expression analysis was conducted three days after drug administration. There was a distinctive pattern, marked by the swift return to normal levels of transcripts related to sJIA overexpressed genes, including genes associated with IL-1 and IL-6 signaling pathways [48]. In another study assessing the dynamics of IL-1RA, IL-18, and IL-6, a reduction in these parameters was noted by the sixth week of canakinumab therapy [49]. Several cohort studies evaluating anakinra therapy have demonstrated consistent improvements in the mentioned cytokines, bringing them back to the normal range throughout the course of treatment [50,51]. However, some studies have demonstrated that IL-18, unlike other pro-inflammatory cytokines and proteins, does not decrease during remission [6].

## 5. Conclusions

We presented study data, attempting to analyze the circulating adaptive immune lymphocyte profile in AOSD patients by assessing the phenotypic characteristics of CD4+ and CD8+ T cells as well as CD19+ B cells, by focusing on the relevant maturation and ‘polarization’ status. T-cell and B-cell subsets were significantly altered in patients with AOSD.

## 6. Limitations

This study has several limitations. Firstly, the study was carried out using a small sample and heterogeneous patient cohort, an inherent limitation of AOSD as a very rare disease. Consequently, we refrained from comparing the clinical patient data with the peripheral blood immune cell profile.

## Figures and Tables

**Table 1:** General characteristics, clinical and laboratory data, treatment of patients with adult-onset Still’s disease during the relapse.

N	Patient 1	Patient 2	Patient 3	Patient 4	Patient 5	Patient 6	Patient 7
Age, years old	28	26	21	63	30	43	52
Sex	2	2	1	1	2	2	2
Years of disease	10	3	1	2	<1	18	6
Course	Polycyclic	Polycyclic	Polycyclic	Polycyclic	Monocyclic	Polycyclic	Polycyclic
Modified Pouchot’s score [14]	5	5	8	4	4	4	2
Laboratory data							
White blood cells × 10^9^/L (4.0–9.0)	9.0	13.1	12.8	17.8	12.8	13.3	6.1
Neutrophils × 10^9^/L (2.0–5.8)	5.7	8.3	10.3	14.9	9.0	9.7	3.8
Neutrophils (%)	63.5	63.2	81.0	84.0	70.0	73.2	62.0
Lymphocytes × 10^9^/L (1.2–3.2)	2.5	3.8	2.3	1.7	3.8	2.1	1.7
NLR	2.28	2.18	4.47	8.76	2.36	4.6	2.23
Platelets (150–400)	411	430	228	394	428	561	215
C–reactive protein, mg/L (0.0–5.0)	117.0	8.2	45.0	51.9	22.6	231.0	6.4
Ferritin, ng/mL (30.0–150)	512	55	1800	1218	311	219	151
ALT, U/L (0.00–33)	12	16	67	36	12	10	14
AST, U/L (5.0–32)	17	17	49	18	12	11	16
Clinical data							
Fever	1	1	1	1	1	1	-
Sore throat	-	1	1	1	-	1	-
Pericarditis	-	1	1	-	-	-	-
Pleuritis	-	1	1	-	-	-	-
Artrhitis	1	1	1	-	1	1	-
Arthralgia	1	1	1	1	1	1	1
Myalgia	1	1	1	-	1	1	-
Rush	1	1	1	1	1	-	1
Lymphadenopathy	1	1	1	1	-	-	-
Splenomegaly	-	-	1	-	-	-	-
Hepatomegaly	-	-	1	1	-	-	-

Treatment on relapse	low dose of NSAIDs	prednisolone 6.25 mg, methotrexate 20 mg	low dose of NSAIDs	low dose of NSAIDs	NSAIDs (ibuprofen 2400 mg) pantoprazol 40 mg	Prednis-olone 10 mg	Prednis-olone 5 mg, metho-trexate 15 mg

Abbreviations: ALT, alanine transaminase; AST, aspartate transaminase; NLR, Neutrophil–Lymphocyte Ratio; NSAIDs, non-steroidal anti-inflammatory drugs; (-)—sign is absent during the relapse.

**Table 2:** Imbalance in circulating Th17 cell subsets in patients with AOSD.

Th17 Cell Subsets	Phenotype	AOSD (n = 7)	Healthy Control (n = 15)	*p* Value
		Med (Q25; Q75)	Med (Q25; Q75)	
% within CM CCR6+ Th17:				
DN Th17	CCR4–CXCR3–	26.51 (20.39; 42.77)	3.27 (2.67; 4.28)	<0.001
class Th17	CCR4+CXCR3–	26.31 (19.32; 30.37)	35.37 (27.72; 42.65)	0.022
Th17.1	CCR4–CXCR3+	16.60 (15.01; 38.03)	32.52 (22.66; 35.55)	0.192
DP Th17	CCR4+CXCR3+	19.11 (10.87; 28.20)	31.39 (26.61;37.05)	0.005
% EM within CCR6+ Th17:				
DN Th17	CCR4–CXCR3–	6.55 (2.94; 11.83)	1.66 (1.53; 3.01)	0.015
class Th17	CCR4+CXCR3–	42.20 (33.62; 46.01)	21.94 (18.65; 25.66)	0.008
Th17.1	CCR4–CXCR3+	23.06 (17.63; 31.09)	47.85 (45.77; 52.17)	<0.001
DP Th17	CCR4+CXCR3+	33.07 (20.28; 34.96)	27.08 (21.61; 31.60)	0.275

**Table 3:** Imbalance in circulating CXCR5-expressing follicular Th cell subsets in patients with AOSD.

Tfh Cell Subsets	Phenotype	AOSD (n = 7)	Healthy Control (n = 15)	*p* Value
		Med (Q25; Q75)	Med (Q25; Q75)	
% CM CCR6+ Tfh:				
Tfh1	CCR6–CXCR3+	29.37 (25.38; 31.79)	27.07 (24.23; 31.27)	0.503
Tfh2	CCR6–CXCR3–	17.00 (11.34; 25.48)	19.23 (14.12; 22.54)	0.698
Tfh17	CCR6+CXCR3–	24.63 (21.75; 27.81)	33.64 (27.26; 37.29)	0.008
DP Tfh	CCR6+CXCR3+	27.31 (18.09; 33.42)	20.76 (18.78; 21.91)	0.130
% EM CCR6+ Tfh:				
Tfh1	CCR6–CXCR3+	25.26 (12.62; 40.94)	22.89 (15.90; 28.48)	0.459
Tfh2	CCR6–CXCR3–	6.94 (1.57; 11.70)	14.23 (9.89; 18.58)	0.010
Tfh17	CCR6+CXCR3–	35.23 (19.69; 46.94)	41.82 (37.10; 48.29)	0.148
DP Tfh	CCR6+CXCR3+	31.53 (22.40; 37.80)	18.86 (17.00; 23.48)	0.007

**Table 4:** Imbalance in circulating B-cell subsets in patients with AOSD.

B Cell Subsets	Phenotype	AOSD (n = 7)	Healthy Control (n = 15)	*p* Value
		Med (Q25; Q75)	Med (Q25; Q75)	
Bm1	IgD+CD38–	9.39 (5.92; 41.21)	7.90 (3.70; 9.88)	0.307
Bm2	IgD+CD38+	50.10 (39.08; 56.04)	60.41 (52.18; 68.75)	0.053
Bm2′	IgD+CD38++	3.73 (0.43; 7.12)	8.21 (5.46; 9.70)	0.045
Bm3 + Bm4	IgD–CD38+++	1.79 (0.26; 4.31)	1.61 (1.37; 2.39)	0.972
eBm5	IgD–CD38+	10.71 (8.66; 13.86)	13.86 (11.27; 17.82)	0.105
Bm5	IgD–CD38–	15.80 (9.89; 19.22)	6.30 (4.54; 8.19)	0.018

## Data Availability

Data is contained within the article and supplementary material.

## Supplementary Materials

The following supporting information can be downloaded at: https://www.mdpi.com/article/10.3390/cimb46020075/s1, Supplementary Table S1: List of monoclonal antibodies for immunophenotyping of peripheral blood CD4+ T-cell subsets; Supplementary Table S2: List of monoclonal antibodies for immunophenotyping of peripheral blood CD8+ T-cell subsets; Supplementary Table S3: List of monoclonal antibodies for immunophenotyping of peripheral blood CD19+ B cell subsets. Supplementary Figure S1: Flow cytometry gating strategy used to define CD4+ T-cell subsets. Supplementary Figure S2: Flow cytometry gating strategy used to define CD8+ T-cell subsets. Supplementary Figure S3: Flow cytometry gating strategy used to define CD19+ B-cell subsets.
