# Supplementary material for: Deep Immunophenotyping of Circulating T and B Cells in Relapsing Adult-Onset Still’s Disease
Source: Curr Issues Mol Biol. 2024 Feb 1;46(2):1177–91. doi: 10.3390/cimb46020075 (PMC10887416; doi:10.3390/cimb46020075)
Supplement: Supplementary file 1 [file cimb-46-00075-s001.zip › cimb-2825065-supplementary.pdf]

Supplementary Table S1. List of monoclonal antibodies for immunophenotyping of peripheral blood CD4+ T cell subsets (all antibodies were manufactured by BioLegend, Inc., San Diego, CA, USA).

N	Antigen	Fluorochome	Clone	Isotype	Cat. number
1	CD45RA	FITC	HI100	Mouse IgG2b, κ	304106
2	CD62L	PE	DREG-56	Mouse IgG1, k	304806
3	CD185 (CXCR5)	PerCP/Cy5.5	J252D4	Mouse IgG1, k	356910
4	CD196 (CCR6)	PE/Cy7	G034E3	Mouse IgG2b, k	353418
5	CD183 (CXCR3)	APC	G025H7	Mouse IgG1, k	353708
6	CD3	APC/Cy7	HIT3a	Mouse IgG2a, k	300318
7	CD4	Pacific Blue	OKT4	Mouse IgG2b, k	317429
8	CD194 (CCR4)	Brilliant Violet 510	L291H4	Mouse IgG1, k	359416

Supplementary Table S2. List of monoclonal antibodies for immunophenotyping of peripheral blood CD8+ T cell subsets (all antibodies were manufactured by BioLegend, Inc., San Diego, CA, USA).

N	Antigen	Fluorochome	Clone	Isotype	Cat. number
1	CD45RA	FITC	HI100	Mouse IgG2b, κ	304106
2	CD62L	PE	DREG-56	Mouse IgG1, k	304806
3	CD185 (CXCR5)	PerCP/Cy5.5	J252D4	Mouse IgG1, k	356910
4	CD196 (CCR6)	PE/Cy7	G034E3	Mouse IgG2b, k	353418
5	CD183 (CXCR3)	APC	G025H7	Mouse IgG1, k	353708
6	CD3	APC/Cy7	HIT3a	Mouse IgG2a, k	300318
7	CD8	Pacific Blue	SK1	Mouse IgG2b, k	344718
8	CD194 (CCR4)	Brilliant Violet 510	L291H4	Mouse IgG1, k	359416

Supplementary Table S3. List of monoclonal antibodies for immunophenotyping of peripheral blood CD19+ B cell subsets (CD45 was manufactured by Beckman Coulter Inc., all other antibodies were manufactured by BioLegend, Inc., San Diego, CA, USA).

N	Antigen	Fluorochome	Clone	Isotype	Cat. number
1	IgD	Alexa Fluor 488	IA6-2	Mouse IgG2a, κ	348216
2	CD38	PE	HB-7	Mouse IgG1, k	356604
3	CD27	PE/Cy7	M-T271	Mouse IgG1, k	356412
4	CD24	Alexa Fluor 647	ML5	Mouse IgG2a, κ	311110
5	CD19	APC/Cy7	HIB19	Mouse IgG1, k	302218
6	CD5	Pacific Blue	L17F12	Mouse IgG2a, k	364024
7	CD45	Krome Orange	J33	Mouse IgG1	B36294

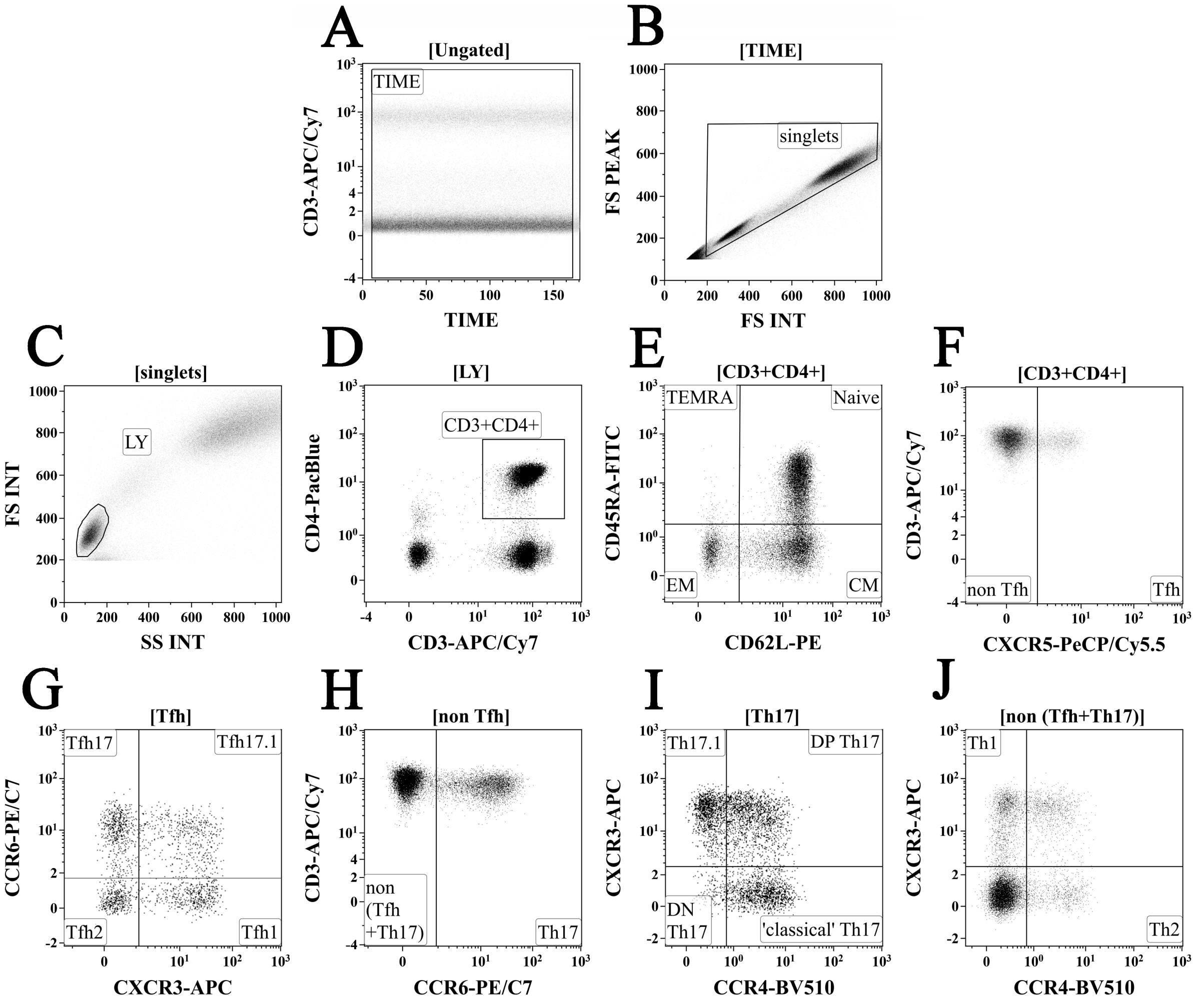

Supplementary Figure S1. Flow cytometry gating strategy used to define CD4+ T cell subsets.

Dot plot A – artifact exclusion by 'time gating'; dot plot B – aggregated cells exclusion; dot plot C – total lymphocyte subset identification; dot plot D – Th cells were identified as CD3+CD4+ subset; dot plot E – within total CD4+ T cells CD45RA+CD62L+ 'naïve' (naïve), CD45RA–CD62L+ central memory (CM), CD45RA–CD62L– effector memory (EM), and CD45RA+CD62L– terminally differentiated CD45RA-positive effector memory (TEMRA) cells were identified. Dot plot F – based on CXCR5 expression follicular Th cells (Tfh) were identified; dot plot G – four main Tfh cell subsets – Tfh1 (CXCR3+CCR6–), Tfh2 (CXCR3–CCR6–), Tfh17 (CXCR3–CCR6+), and DP Tfh (CXCR3+CCR6+) were determined within total Tfh cells. Dot plot H – Th17 cells were detected within CXCR5 negative Th cell, and, dot plot I – four main Th17 cell subsets, including 'classical' Th17 cells with CXCR3–CCR4+ phenotype, double-negative (DN) Th17 with CXCR3–CCR4– phenotype, double-positive (DP) Th17 cells with CXCR3+CCR4+ phenotype, and Th17.1 cells with CXCR3+CCR4– were determined within total Th17 cells. Finally, dot plot J – within CXCR5–CCR6– T cell subset we determined Th1 (CXCR5–CCR6–CCR4–CXCR3+) and Th2 (CXCR5–CCR6–CCR4+CXCR3–) cell subsets.

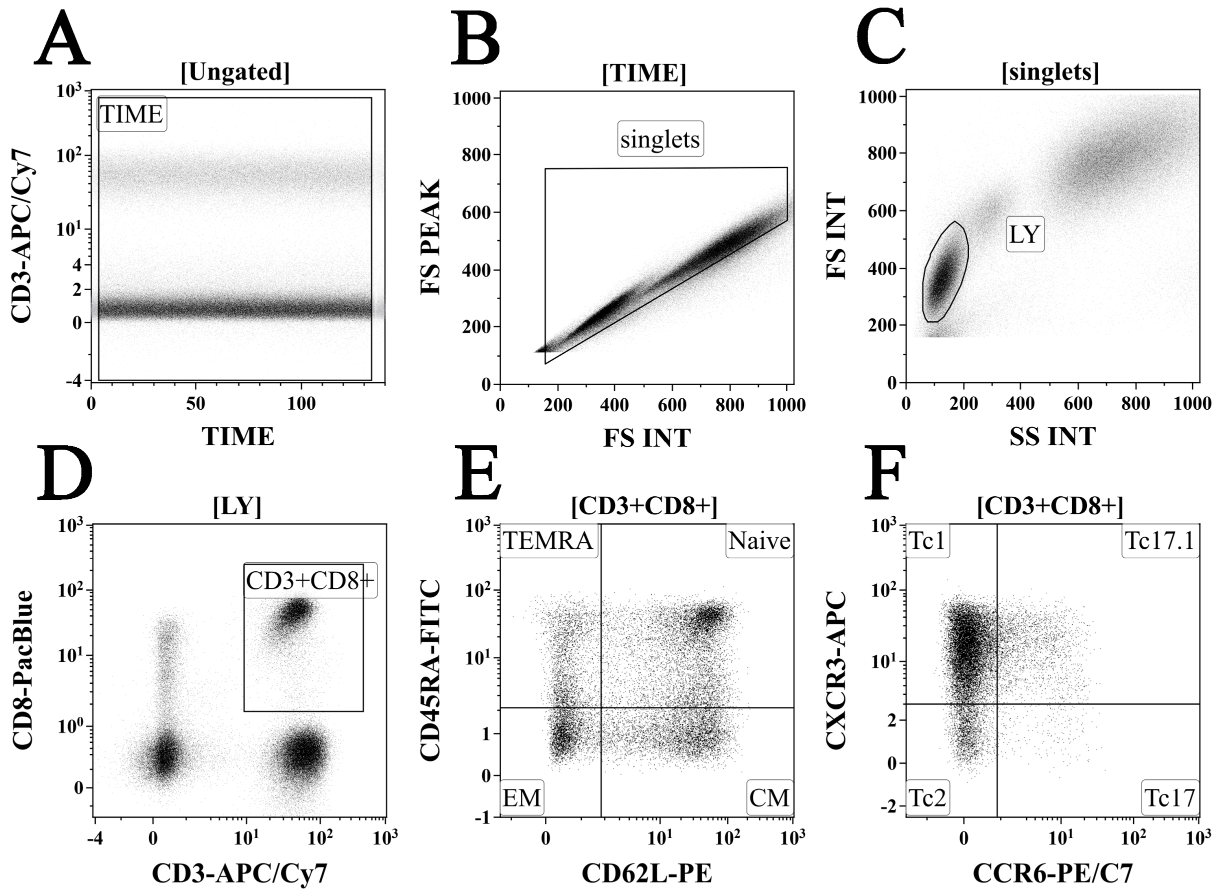

Supplementary Figure S2. Flow cytometry gating strategy used to define CD8+ T cell subsets.

Dot plot A – artifact exclusion by 'time gating'; dot plot B – aggregated cells exclusion; dot plot C – total lymphocyte subset identification; dot plot D – CD8+ T cells were identified as CD3+CD8+ subset; dot plot E – within total CD8+ T cells CD45RA+CD62L+ 'naïve' (naïve), CD45RA–CD62L+ central memory (CM), CD45RA−CD62L− effector memory (EM), and CD45RA+CD62L− terminally differentiated CD45RA-positive effector memory (TEMRA) cells were identified. Dot plot F – within total CD8+ T cells four main 'polarized' subsets were determined, including CCR6−CXCR3+ Tc1, CCR6−CXCR3− Tc2, CCR6+CXCR3− Tc17, and double-positive CCR6+CXCR3+ Tc17.1 cells.

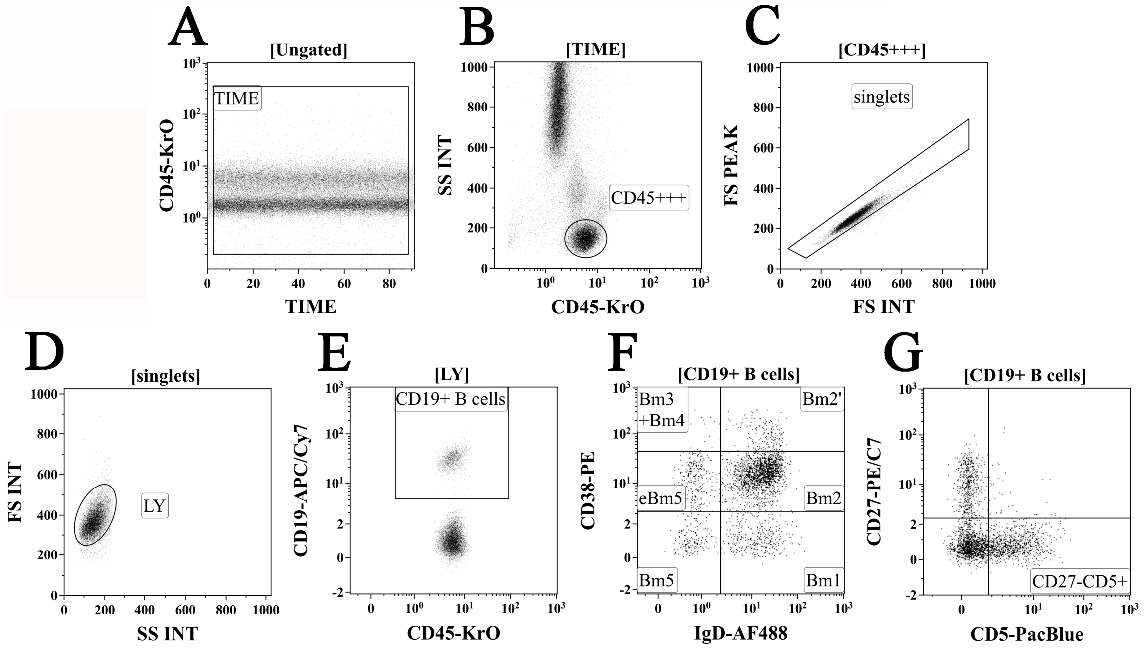

Supplementary Figure S3. Flow cytometry gating strategy used to define CD19+ B cell subsets.

Dot plot A – artifact exclusion by 'time gating'; dot plot B – lymphocytes were determined as CD45+++SSlow cells; dot plot C – aggregated cells exclusion; dot plot D – cell debris exclusion; dot plot E – CD19+ B cells were identified based on CD19 expression. Dot plot F – so-called 'Bm1-Bm5' classification, that allowed us to determine main six B cell maturation subsets, including IgD+CD38– 'virgin naïve' Bm1 cells, IgD+CD38+ "activated naïve" Bm2 cells , pre- IgD+CD38++ pre-germinal-centre Bm2' cells, IgD–CD38++ 'Bm3 + Bm4' subset, containing centroblasts and centrocytes, IgD–CD38+ early memory eBm5, and IgD–CD38– resting memory cells Bm5 cells. Dot plot G – CD5+CD27– B cell determination within total B cell subset.
